# Particulate Production and Composite Dust during Routine Dental Procedures. A Systematic Review with Meta-Analyses

**DOI:** 10.3390/ma13112513

**Published:** 2020-05-31

**Authors:** Anna Iliadi, Despina Koletsi, Theodore Eliades, George Eliades

**Affiliations:** 1 Department of Biomaterials, School of Dentistry, National and Kapodistrian University of Athens, 11527 Athens, Greece; annaeliades@gmail.com (A.I.); geliad@dent.uoa.gr (G.E.); 2 Clinic of Orthodontics and Pediatric Dentistry, Center of Dental Medicine, University of Zurich, 8032 Zurich, Switzerland; d.koletsi@gmail.com

**Keywords:** composite dust, nanodust, nanoparticle, composite grinding, composite polishing, orthodontic debonding, composite restoration, airborne dust, aerosolized particles, dental practice

## Abstract

Composite dust generation is most likely a continuous and daily procedure in dental practice settings. The aim of this systematic review was to identify, compile and evaluate existing evidence on interventions and composite material properties related to the production of aerosolized dust during routine dental procedures. Seven electronic databases were searched, with no limits, supplemented by a manual search, on 27 April 2020 for published and unpublished research. Eligibility criteria comprised of studies of any design, describing composite dust production related to the implementation of any procedure in dental practice. Study selection, data extraction and risk of bias (RoB) assessment was undertaken independently either in duplicate, or confirmed by a second reviewer. Random effects meta-analyses of standardized mean differences (SMD) with associated 95% confidence intervals (CIs) were employed where applicable. A total of 375 articles were initially identified, resulting in 13 articles being included in the qualitative synthesis, of which 5 contributed to meta-analyses overall. Risk of bias recordings ranged between low and high, pertaining to unclear/raising some concerns, in most cases. All types of composites, irrespective of the filler particles, released significant amounts of nano-sized particles after being ground, with potentially disruptive respiratory effects. Evidence supported increased % distribution of particles < 100 nm for nanocomposite Filtek Supreme XTE compared to both conventional hybrid Z100MP (SMD: 1.96, 95% CI: 0.85, 3.07; *p*-value; 0.001) and nano- hybrid Tetric EvoCeram (SMD: 1.62, 95% CI: 0.56, 2.68; *p*-value: 0.003). For cytotoxicity considerations of generated aerosolized particles, both nanocomposites Filtek Supreme XTE and nanohybrid GradiO revealed negative effects on bronchial epithelial cell viability, as represented by % formazan reduction at 330–400 μg/mL for 24 hours, with no recorded differences between them (SMD: 0.19; 95% CI: −0.17, 0.55; *p*-value: 0.30). Effective and more rigorous management of dental procedures potentially liable to the generation of considerable amounts of aerosolized composite dust should be prioritized in contemporary dental practice. In essence, protective measures for the clinician and the practices’ personnel should also be systematically promoted and additional interventions may be considered in view of the existing evidence.

## 1. Introduction

Dental resin composites are currently used for a wide spectrum of preventive and restorative procedures such as sealants, restorations of carious lesions, aesthetic restorations, core build ups, indirect restorations, as well as bracket bonding in orthodontics and attachment configuration in aligner treatment [1]. Due to their strength and ability to mimic the optical characteristics of enamel and dentin, they have been considered as the forefront representatives of material science in clinical dentistry [2].

Resin composites are normally composed of a resin matrix, inorganic filler particles and a coupling agent to bond the filler to the matrix [3]. The filler particles improve the physical and mechanical properties of the composite [4]. Furthermore, they reduce the thermal expansion coefficient, polymerization shrinkage, water sorption and solubility, improve surface properties and handling [4]. For radiopacity, fillers of metal oxides are added such as of barium, strontium, zinc, aluminum or zirconium [5]. Inorganic filler particle size distributions usually are below 0.4 μm to guarantee surface gloss retention. In several products, nano-sized particles are used exclusively or are added to fill the interparticle spaces of blended particle sizes, thus optimizing filler packing [3]. An increase in the volume percentage of nano-fillers results in superior restoration surface finishing and the likelihood of the material’s biodegradation diminishes [6].

During routine dental procedures, such as surface finishing and polishing, removal of old composite restorations, preparation of core build up for crown restoration and bracket debonding in orthodontics, dentists and dental personnel may leave themselves exposed to and/or inhale aerosolized composite dust on a daily basis [7]. The breakdown content of composites comprises micro-fragments as well as single filler particles that may bear the potential to penetrate deep into the lungs surpassing the natural respiratory defense mechanism of mucus and cilia [8]. Existing studies have revealed that chronic inhalation of respirable dust and nano-particles may incite local and systematic toxicity when absorbed in the blood or the lymph system [9], or even provoke more serious conditions such as pneumoconiosis [10].

Thus the broad aim of this systematic review was to collectively appraise the existing evidence on interventions and material properties related to aerosolized composite dust production in dental practice or in simulated environment, through standard dental procedures. The null hypothesis formulated was that there is no significant amount of respirable composite dust produced after grinding practices and variations in intervention procedures related to grinding instrumentation or differences in material conformation do not result in a differential content of dust production, or alternate findings regarding cytotoxicity and safety.

## 2. Materials and Methods

### 2.1. Protocol, Registration and Reporting

The protocol of this study was registered with the Open Science Framework [11] (https://osf.io/st9mx/). Reporting was conducted in accordance to the PRISMA guidelines [12,13].

### 2.2. Eligibility Criteria

Eligibility criteria for study selection were schemed as follows:

—Study design: randomized controlled trial (RCT), prospective clinical trial (non- randomized), retrospective/prospective cohort, in-vitro, laboratory studies, irrespective of the groups under comparison.

—Participants: patients (no specified age) undergoing routine dental procedures engaged to composite treatment, or orthodontic fixed appliance debonding/aligners’ attachment removal. For in vitro/pre-clinical research, any type of procedure simulating in vivo practice was considered.

—Intervention: any type of routine dental procedure with the involvement of composite grinding in vivo, or simulating alternative.

—Comparator: any type of routine dental procedure as comparison, involving composite grinding, in vivo or simulating; subgroups of various types of composites, mainly based on filler particles were also be eligible.

—Outcome(s): including but not confined to composite dust release, particulates or (nano)-particles release concentration and byproducts, toxicity, cytotoxicity.

—Exclusion criteria: case studies, animal studies.

### 2.3. Search Strategy and Study Selection

Electronic searching was employed within 7 databases including published and unpublished research, with no language restriction or other filter modifications, on 27 April 2020 (Appendix A). The respective databases searched were: Medline via Pubmed, Scopus, Cochrane Central Register of Controlled Trials (CENTRAL), Cochrane Database of Systematic Reviews (CDSR). Moreover, unpublished literature was searched in the Open Grey, the ClinicalTrials.gov (www.clinicaltrials.gov), the National Research Register (www.controlled-trials.com). A manual search of the eligible for inclusion articles was employed for any additional potential inclusion and authors of the included papers were contacted when in need to clarify on data extraction or data curation. Keywords involved “composite dust”, “suspended particle”, “aerosol”, “nano-dust”, “grinding”, “polishing”.

### 2.4. Data Collection

Data extraction was implemented in pre-piloted standardized forms by a single reviewer (AI), not blinded to study origin or author identity, while all entries were confirmed by a second investigator (DK). Specifically, information entries were related to study identity, study design, sample size, intervention, comparators, outcomes, technical information for laboratory studies and method of analysis.

### 2.5. Risk of Bias in Individual Studies

Risk of bias assessment was performed independently and in duplicate by two authors (AI, DK). Any disagreements were settled after consultation with a third author (TE). For included RCTs, the updated Cochrane RoB 2.0 tool was used [14]. A modification of this tool was utilized for the included in vitro or small in vivo studies, as no pre-determined guidelines to assess the risk of bias exist and in order to incorporate specific important elements that would help identify the presence of potential bias. These include selection bias [15,16], performance bias [17], attrition bias and reporting issues [18,19].

### 2.6. Summary Measures and Data Synthesis

Prior to any decision to quantitatively pool data from individual studies, clinical heterogeneity was examined in terms of individual study settings, trial or laboratory conditions, inclusion criteria or methods of analyses. If applicable, statistical heterogeneity was also planned to be examined, first visually, through inspection of the confidence bounds within the forest plots, as well as statistically, as indicated by a *p*-value below the level of 10% for the test (*p* < 0.10). An *I*^2^ test for homogeneity was also planned to be undertaken. 

Random effects meta-analyses were planned as they were considered more appropriate to incorporate individual study findings if applicable. In view of the anticipated continuous nature of the expected outcomes, treatment effects were calculated through pooled standardized mean differences (SMDs) or weighted mean differences (WMD) with associated 95% confidence intervals (95% CIs).

### 2.7. Risk of Bias Across Studies

If more than 10 studies were included in meta-analyses, publication bias was planned to be explored through standard funnel plots and Egger’s regression test [20].

## 3. Results

### 3.1. Search Details

**Figure 1:**
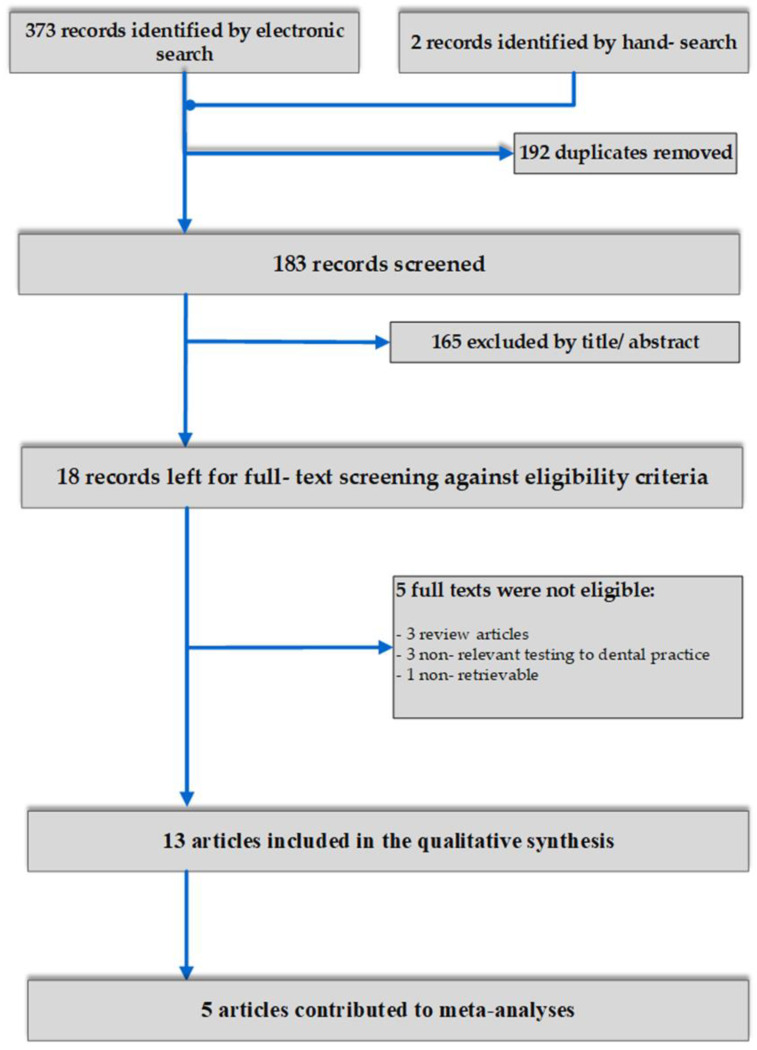
Flow-diagram of study selection.

A total of 375 papers were initially retrieved after application of all search strategies across the array of the databases. After duplicate removal and screening per title and abstract, 18 articles were left for full text assessment, while 13 studies were eligible for inclusion in the qualitative synthesis [1,21,22,23,24,25,26,27,28,29,30,31,32]. Of those, 5 contributed to meta-analyses across different comparisons and outcomes [1,23,25,26,31]. (Figure 1).

### 3.2. Study Design and Characteristics

Table 1 presents the detailed description of the included studies, pertaining to study design, interventions, comparators, technical details and outcomes.

The studies included cover a period of 17 years, from 2003 and on, with the majority of the studies being published the last decade (9/13; 69%), while 7 of those studies have appeared in the literature over the last 5 years (7/13; 53.8%). Study design was predominantly in vitro, pertaining to laboratory studies simulating clinical conditions for composite grinding [1,21,22,23,24,25,26,27,29,30,31]. One RCT was identified [32] as well as one small cohort study [28]. In addition, two in vitro studies reported on a limited scale clinical application of their work [1,31].

Five of the included studies were designed to assess composite dust generated in conditions of enamel clean-up after orthodontic fixed appliances removal [22,27,28,29,32], while the rest reported on grinding composite blocks or sticks in the lab, simulating restorative dental procedures [1,21,23,24,25,26,30,31]. The latter mainly pertained to the comparison of different types of composites based on the filler particles of each and were mainly represented by nanocomposites, nano-hybrid, micro-hybrid and/or conventional hybrid composites ground under rough diamond or carbide tungsten burs with the use of a micromotor and under dry conditions. The former set of studies were related to reported comparisons on different types of orthodontic brackets after debonding, examined effects of different curing procedures and also effect of speed of handpiece in use, presence of water cooling, facemask, or high-volume evacuator (HVE). Sample size blocks ranged from 3 to 20 per examined group. All related outcomes examined pertained to a set of two major assemblies; first, particulate concentration, number and size of the produced dust and second, estrogenic and cytotoxic effects of the aerosolized dust and monomer or bisphenol-A (BPA) release (Table 1).

More technical data on the instrumentation used by the respective studies to achieve the assessed outcomes are presented in Table 1 and in summary these include an array of laboratory equipment: scanning mobility particle sizer (SMPS), transmission electron microscopy (TEM), scanning electron microscopy (SEM) and energy-dispersive X-ray spectroscopy (EDX), Fourier transform infrared spectroscopy (FTIR), diffusion size classifier (DiSi) and others.

### 3.3. Risk of Bias within Studies

Overall risk of bias assessment of the included studies revealed unclear to high risk of bias. Specifically, for the in vitro studies [1,21,22,23,24,25,26,27,29,30,31] the most concerning domains were blinding of the outcome assessors, where none reported on its implementation and also risk of reporting bias, since none ascertained on whether a pre- registered protocol for the designed study existed. In contrast, baseline similarity of the examined groups was considered fairly adequate (Table 2). The small cohort study [28] was prone to high risk of bias, due to the single arm design (Table 2), whereas the sole RCT [32] raised some concerns as well, regarding ascertaining the measurement of the outcome as well as the possibly non-existent pre-formulated protocol, since no study registration could be detected. On the other hand, research domains such as randomization and concealment of allocation to treatment groups, or deviations from the prescribed interventions and losses to follow-up were adequately described (Table 3)

### 3.4. Effects of Interventions, Meta-Analyses, Additional Analyses

Overall, five of the included studies [1,23,25,26,31] contributed to the quantitative mathematical syntheses across all identified outcomes. Quantitative data for all relevant meta-analyses, as well as for single study findings, where applicable, are shown in Table 4. 

**Figure 2:**
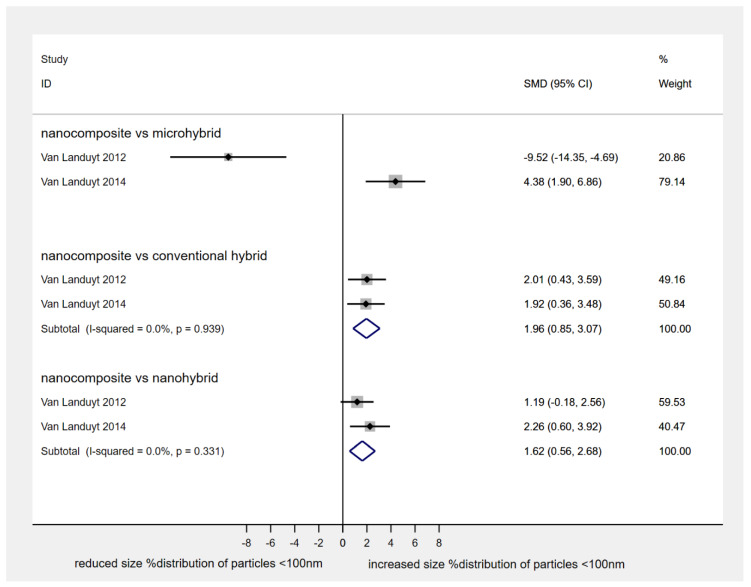
Standardized mean differences in size % distribution of particles < 100 nm. (nanocomposite, Filtek Supreme XTE; microhybrid, Gradia Direct; conventional hybrid, Z100 MP; nanohybrid, Tetric EvoCeram).

**Figure 3:**
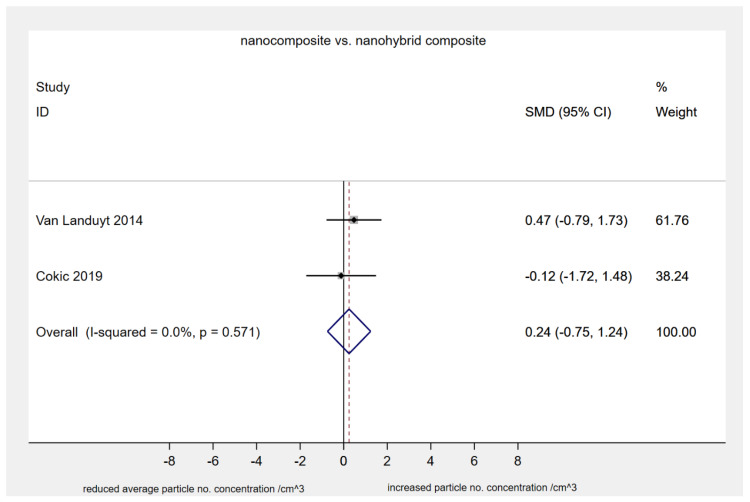
Standardized mean differences in average particle number concentration (#/cm^3^ × 10^6^), (nanocomposite, Filtek Supreme XTE; nanohybrid, GradiO).

**Figure 4:**
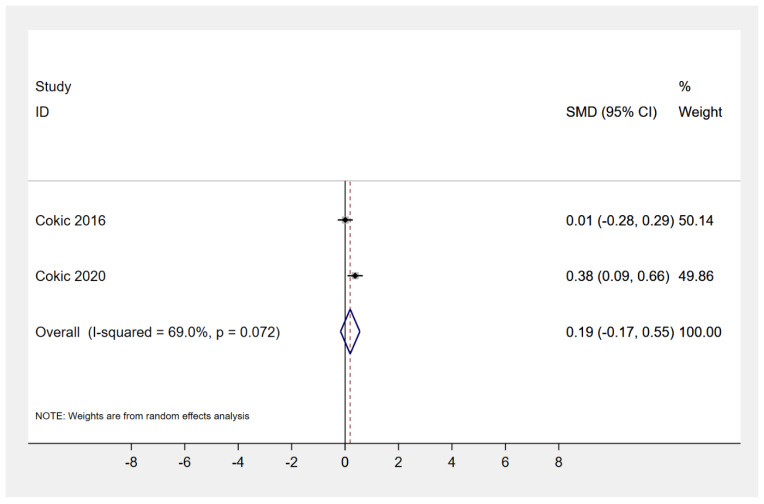
Cell viability by WST-1 assay, represented by % formazan reduction at 330–400 μg/mL (nanocomposite, Filtek Supreme XTE; nanohybrid, GradiO).

When size% distribution of nano-sized particles (i.e., < 100 nm) identified after composite grinding in air samples was assessed, the nanocomposite Filtek Supreme XTE produced a higher number of those compared to both conventional hybrid Z100MP (2 studies, SMD: 1.96, 95% CI: 0.85, 3.07; *p*-value: 0.001) and nano-hybrid Tetric EvoCeram (2 studies, SMD: 1.62, 95% CI: 0.56, 2.68; *p*-value: 0.003) (Figure 2). However, the average particle number concentration (in/cm^3^ × 10^6^) detected after grinding of either nanocomposite Filtek Supreme XTE or nanohybrid GradiO was similar (2 studies, SMD: 0.24; 95% CI: −0.75, 1.24; *p*-value: 0.63) (Figure 3). Interestingly, all types of restorative composites (i.e., nano-, nanohybrid, microhybrid, hybrid) released significant amounts of nanoparticles with a median diameter varying within the range of 38 to 70 nm during grinding, with a potential to exhibit agglomerating dynamic only a few minutes after the procedure (as measured for up to 7 minutes post-procedurally). On the same lines, efforts to assess cytotoxicity of produced particles, through estimation of human bronchial epithelial cell viability, as represented by % formazan reduction at 330–400 μg/mL for 24 hours, revealed mild but gradual reduction for both nanocomposites Filtek Supreme XTE and nanohybrid GradiO (2 studies, SMD: 0.19; 95% CI: −0.17, 0.55; *p*-value: 0.30) (Figure 4). 

Likewise, findings from a single study [26] reported on cytotoxicity and genotoxic behavior of ground composite dust with the additional assessment of an orthodontic composite containing quartz particles (Transbond XT). Results revealed significantly higher cytotoxic and genotoxic activity of the orthodontic adhesive air-sampled particulates, detected on exposed human bronchial epithelial cells; this was evident against both Filtek Supreme XTE nanocomposite (% formazan reduction at 400 μg/mL: MD: 6.8; 95% CI: 4.7, 8.9; *p*-value < 0.001; % cell membrane integrity reduction: MD: 6.1; 95% CI: 2.5–9.7; *p*-value: 0.001), as well as GradiO nanohybrid composite (% formazan reduction at 400 μg/mL: MD: 10.6; 95% CI: 7.3, 13.9; *p*-value < 0.001; % cell membrane integrity reduction: MD: 10.9; 95% CI: 7.8, 14.0; *p*-value < 0.001). Dust from Transbond XT was also identified as being disruptive of the initial stages G0/G1 of the bronchial epithelial cells’ cycle, though exhibiting a negative effect on cells’ growth potential (Table 4).

Additional evidence on estrogenicity of orthodontic composites’ generated dust after simulated orthodontic debonding and enamel clean-up, with different curing methodologies, comes also from a single study back in 2009 [27]. Findings suggest an increased human breast adenocarcinoma proliferating induction capacity of eluents containing airborne composite particulates, for both chemically cured (CC, System 1+: MCF-7 cell proliferation %control: MD: 60; 95% CI: 51.5, 68.5; *p*-value < 0.001) as well as light-cured (LC, Blugloo: MCF-7 cell proliferation %control: MD: 28; 95% CI: 22.4, 33.6; *p*-value < 0.001) composites, compared to normal saline (Table 4). On the same grounds, evidence from a single study [24], revealed release of BPA, which has been identified as an endocrine and potentially estrogenic disruptor, from composite nanodust. This was also supplemented by detection of methacrylate monomer release in the dust, irrespective of the type of composite but corresponding to the composite’s composition. 

The report of a single study [25] on the effect of water cooling after slow-speed handpiece usage for restorative practice, revealed increased average particle number concentration in absence of water (example: MD for Filtek Supreme XTE: 1.5; 95% CI: 1.2, 1.7; *p*-value < 0.001) (Table 4). This effect was prevalent for all tested adhesives.

On this respect, an earlier report by Johnston et al, in 2009 [29], structured on non- parametric data, revealed a significant difference in particulate production (mg/m^3^), between a slow-speed handpiece without water cooling and a high-speed handpiece with cooling water, both using a tungsten carbide bur, after composite grinding following orthodontic debonding of metal brackets. The high-speed handpiece scored higher (*p*-value < 0.001). Further, use of surgical facemask significantly reduced the amount of detectable particles overall (*p* < 0.001), while a high-volume evacuator (HVE) did not appear very effective.

Lastly, the findings of the sole RCT contributing to the present systematic review [32] did not reveal any differential effect of bracket type (either flash-free adhesive coated or conventional ceramic) on the amounts particulates produced during enamel clean-up after debonding. 

### 3.5. Risk of Bias across Studies

Publication bias and small- study effects could not be explored, in view of the limited number of studies included across identified outcomes eligible for data pooling.

## 4. Discussion

### 4.1. Findings in Context

Particulate generation during routine procedures in dental practice is one of the major concerns for clinical dentistry and one that contributes knowledge to the general notion regarding potentially hazardous aerosolized material after application of certain procedures in everyday clinical practice. Prior investigations have highlighted the pathogenic load of bio-aerosols in dentistry [33], or undertaken endorsements to identify potentially effective measures against bio-aerosol content from a microbiologic perspective [34]. This becomes especially alarming under the light of the severe acute respiratory syndrome–coronavirus-2 (SARS-CoV-2) pandemic [35], where healthcare workers are in the frontline of suspended droplet or aerosol-related contamination and inhalation/respiration protection measures are crucial [36]. Acute or chronic health effects of airborne particles of the ultrafine and nanoscale fraction, in general, have long been investigated and reported [37,38]; however, their impact on dentistry and healthcare workers related to dental practice is still not known and potentially ignored [39].

Certainly, the findings of the present review dictate the rejection of the null hypothesis, based on pooled as well as single study findings. These reveal a considerable amount of particles of the respiratory fraction, but mainly nanosized particles, being produced irrespective of the handpiece instrumentation, the presence or absence of water cooling, or the particular dental procedure involving composite grinding under evaluation. In addition, variations across studies, favoring or opposing certain materials and/or procedures were detected.

Focus on aerosolized composite dust generation has been three-fold: first, composite composition and characterization of the produced dust; second, type of outcome induced after potential inhalation or respiration, pertaining to cytotoxicity or potential deposition of particles and their relative dynamic to go deep into the respiratory tract, especially interacting with the tracheobronchial and alveolar region of the tract and finally the type of composite grinding in terms of the handpiece instrumentation method used and speed round of the handpiece, as well as in terms of the water presence or otherwise. Elemental analysis of airborne dust after composite grinding revealed the presence of mostly, silica (Si), aluminium (Al) and barium (Ba), while traces of other elements were also detectable in the produced powders. Variations exist between individuals on the composition, amount and quantities of particulates deposited in certain regions of the tract, with deposition amounts and areas being dependent on particulate diameter, air movement, or even breathing rate [40]. It has been argued that although a considerable amount of novel composites used in clinical practice are accountable for nano-particle fillers (<100 nm), this does not necessitate an inductive assumption for a similarly sized and amounted particulates generated during grinding of the material, that also interact with the respiratory tract membranes. In essence, it is largely unknown whether nanoparticles produced during grinding procedures originate from nano-filler compounds of the composites or are parts of larger particles being ground. Friction heat through grinding instrumentation routines has been considered liable for composite matrix thermal decomposition, mechanical aging, increase in the C=C conversion of bonds on surface material and finally air suspension of dust [21,27,31,41,42], with a median range of dimension of 38 to 70 nm [31]. Based on current evidence, research has been mostly carried forward though identification of inhalable and respirable aerosolized particles, with particle sizes of lower than 5 μm, but more specifically around 10 nm being of most concern, due to their capacity to surpass standard cilia and mucus human respiratory defense system [1,8]. Particular concerns have been raised about such dimensions of nanoparticles, due to their increased surface to volume ratio, which renders them highly reactive when implicated with a cellular interface, as compared to counterparts of larger particle sizes [39]. In this respect, alarming findings have emerged from a recent study [26], regarding the cytotoxic and potential genotoxic effect of airborne dust produced by an orthodontic adhesive, on bronchial epithelial cells. It has been argued that this might be attributed, at least partly, to the sub-micron crystalline quartz particle composition of the adhesive, as quartz has long been identified as particularly reactive, oxidative and potentially toxic compound [43]. This was not the first study on orthodontic adhesives and reactivity with human cells. A previous report [27], has revealed an estrogenic dynamic of both chemically as well as light cured orthodontic adhesives, in particular under simulated orthodontic debonding setting. To this end, further research in the field is certainly crucial.

Based on theoretical grounds and some perspectives, water cooling during composite grinding has been proposed, as a measure that could potentially contribute to the formulation of increased particle size, with nano-sized dust particulates being trapped to larger water droplets, thus offering enhanced protection against nano-particle respiration and penetration of sensitive human organs [1,31]. However, one should effectively consider the potential for generation of pronounced aerosol in dental settings, when water cooling practices are followed, as well as the possibility of increased pathogen diffusion via this route [34]. Additionally, research in the field of orthodontics revealed an increased amount of particulate production after high-speed, water supplemented adhesive grinding following fixed appliance therapy [29]. In any case, a trade- off between aforementioned routines is anticipated, based on patients’ needs in addition to patients’, doctor’s and personnel’s safety, with further evaluation of the timing and duration of the procedure.

Realistic management of all dental procedures that generate aerosols in everyday practice, in the era of a pandemic [35], is more pertinent than ever. Given this, minimization of composite dust production is anticipated. Such practices may involve substitute procedures, which may be used as proxies to the widely used material grinding protocols, when feasible. An example pertaining to orthodontic debonding routines for enamel clean-up after fixed appliance removal, might be to target a carefully selected bracket-to-adhesive interface that would eliminate composite remnants at debonding or better, induce a cohesive resin fraction of the bulk of the composite upon debonding, thus, making simple scaler removal or the remnants practicable, without use of an air-turbine or micromotor handpiece [44]. Other concepts may involve reduction of large-scale composite attachment use for aligner orthodontic therapy, by evading adoption of company preset determination of attachments’ distribution, coupled with careful selection of patients and malocclusions that may be eligible for low-scale attachment- aligner therapy. Grinding and polishing procedures of restorative treatments should also be minimized, by careful pre-polymerization sculpturing of the composites. A two-stage procedure, for example involving laboratory preparation of composite in-/on-lays, would also be a viable alternative, when practicing on demanding restorative treatments, which may entail a considerable amount of post- polymerization grinding and/or polishing. Additional to that, in- house measures of self- protection should be considered both for the dentists as well as for the clinic personnel. Surgical masks with filter protection layers and small particle filtration efficiency, appropriate ventilation and evacuation instrumentation are recommended.

### 4.2. Strengths and Limitations

This is the first comprehensive report that collectively appraised the evidence from in vitro simulated or clinical reports on particulate generation and dust production after composite grinding under a range of simulated dental procedures. In addition, based on the available evidence, a limited documentation on clinical condition settings was also possible. The review was registered a priori, to ensure against reporting shortcomings and a large-scale search on 7 electronic databases of both published and unpublished literature, in addition to a manual search was also employed. Methodology and reporting was sound and robust, following current state of the art guidelines [12,13].

Limitations exist as well. These are framed across eligible studies’ inherent characteristics of content and internal validity. Evidence revealed a certain need to train scientists working on laboratory research to formulate a priori registered protocols, safeguarding against emerging reporting and dissemination issues; furthermore, detection bias was an additional concern. Researchers involved in outcome assessment should act independently and without prior knowledge on the study protocol, otherwise masking, if possible, would be a viable solution [18,19].

A quantitative synthesis was possible on as limited scale, thus offering a restricted amount of precision to the estimated effects, but this was still dependent on the abundance of original research, as well as on the homogeneity of included studies, materials, interventions and outcomes, or otherwise [45,46].

## 5. Conclusions

Aerosolized composite dust should be acknowledged as an additional occupational hazard in dental practice. Procedures with implicated potential to generate airborne nanosized particles should be employed with caution or minimized to a realistic extent, while taking of protective measures of the operating clinicians as well as the practice’s personnel are not to be neglected, even in limited-duration procedures. Certainly, compilation of additional evidence constitutes a necessity to further work on the endorsement of safety guidelines for clinical practice.

## Figures and Tables

**Table 1:** Characteristics of included articles (*n* = 13, in alphabetical order).

Study ID	Sample/Participants	Intervention (one or > 1)	Comparator (one or >1)	Technical Data	Outcome(s)
Bradna et al, 2017 In vitro Setting: composite specimens in Teflon molds	Composite grinding (no water cooling); air sampling 30 sec after end of grinding, closed cabinet (2 × 1.3 × 2.5 m) 4 composites	Filtek Ultimate (mixture of primary SiO_2_ and ZrO_2_ nanoparticles and zirconia- silica agglomerates) Estellite Sigma Quick, Supra-nano fill, silica-zirconia	Charisma Microhybrid (with Ba glass microparticles) Unfilled resin (LC Varnish)	Diamond round bur (medium and fine)/tungsten carbide (20 blade) Assessment: on-line spectrometer	Aerosol particle concentration Particle size distribution
Day et al, 2008 In vitro Setting: extracted teeth/impactor simulating lung	24 teeth; orthodontic debond procedures; air sampling 15 min, at 30 cm sampling distance	High speed handpiece (water +/-)	Slow speed handpiece (water +/-) Control; no procedure	Spiral fluted tungsten carbide bur; Transbond XT adhesive and primer; Assessment: SEM, X-ray	Particulate matter Particulate composition
Cokic et al, 2016 In vitro Setting: composite sticks in metal mold	Composite grinding (no water cooling); laminar flow cabinet and dust transferred for to cell culture for toxicity assessment (96-well plates) 4 types composites	Filtek Supreme XTE nanocomposite Grandio and Tetric nanohybrid	Grandia Direct Microhybrid Z-100 MP conventional hybrid	All samples ground with rough diamond bur (100 μm) (micromotor, 200.000 rpm) Assessment: WST-1 assay; LDH leakage assay	Cytotoxicity against human bronchial cells IL-1β, IL-6 cytokine release Characterization of composite particles/dust
Cokic et al, 2017 In vitro Setting: composite sticks in metal mold	Composite grinding, (no water cooling), plexiglass chamber 27 × 27 × 42 cm; air sampling immediately before grinding until 10 min thereafter 4 types of composites (n = 5/per composite)	Filtek Supreme XTE nanocomposite Grandio (nanohybrid composite)	Gradia Direct Microhybrid Z100 MP conventional hybrid	All samples ground with rough diamond bur (100 μm) (micromotor, 200.000 rpm) Assessment: LC–MS/MS, TEM-EDS	Release of methacrylate monomers and BPA in water and ethanol Ultra-morphological and chemical analysis of dust
Cokic et al, 2019 In vitro Setting: composite sticks in metal mold	Composite grinding, closed chamber 1 m^3^; measurement 3 min during grinding and additional 10 min water +/- 7 types of composites (n = 3/per composite)	Filtek Supreme XTE nanocomposite GrandioSO and Herculite XRV Ultra nanohybrid composite	Spectrum TPH3 micromatrix with nanotechnology Herculite XRV microhybrid Durafill VS microfilled anterior composite Heliomolar flow microfill, flowable Control: no composite grinding	All samples ground with rough diamond bur (100 μm) (micromotor, 200.000 rpm) Assessment: SMPS, TEM-EDS	Particle number concentration, particle size distribution and average particle size Ultra-morphological and chemical analysis of dust
Cokic et al, 2020 In vitro Setting: composite sticks in metal mold	Composite grinding (no water cooling), plexiglass chamber 27 × 27 × 42 cm; measurement 3 min during grinding and additional 10 min; dust transferred for to cell culture for toxicity assessment (96-well plates)	Filtek Supreme XTE nanocomposite Grandio nanohybrid composite	Transbond XT	All samples ground with rough diamond bur (100 μm) (micromotor, 200.000 rpm) Assessment: DLS/ELS, WST-1 assay, LDH assay, comet assay, TEM	Particle size distribution Cell viability Membrane integrity DNA damage in individual cells Cellular uptake of particles by epithelial cells
Gioka et al, 2009 In vitro Setting: composite applied to cellulose-covered brackets	Composite grinding in simulated debonding, in a glass tube; no water cooling (n = 20/per composite)	Blugloo light cure	System 1 + chemical cure	All samples ground with 8-fluted tungsten carbide bur; high speed handpiece Assessment: micro-ATR FTIR spectroscopy, Scanning electron microscopy, X-ray microanalysis	Molecular characterization of particles Morphologic condition and structure Elemental composition of particles Estrogenicity
Ireland et al, 2003 In vivo (small cohort) Setting: dust collection during debonding in patients	Enamel clean up after debonding, air sampling 5–10 min	Transbond XT Ketac-Cem (glass polyalkenoate cement)-for bands	none	spiral fluted tungsten carbide bur; slow-speed handpiece Assessment: SEM, EDX	Particle size Chemical composition
Johnston et al, 2009 In vitro Setting: debonding of extracted teeth/impactor simulating lung	Enamel clean up after debonding; total sampling time 20 min (n = 20/group)	Stainless steel brackets, slow/high handpiece, water +/-, surgical facemask +/-, HVE +/-	Ceramic brackets, slow handpiece, no water, no facemask, no HVE. Fractured ceramic, high and slow handpiece, water +/-, surgical facemask +/-, HVE +/-	Carbide spiral fluted bur (ss, ceramic brackets) Diamond fissure bur + tungsten carbide bur (fractured ceramic brackets) Adhesive for brackets: Transbond XT Assessment: SEM, EDX	Qualitative/ quantitative analysis of particle size and composition
Nilsen et al, 2019 In vitro Setting: restorative treatment on phantoms	Restoration polishing; sampling period from the start of bonding procedure sampling pumps + water collection	Ceram.x universal	Clearfil SE Bond (primer) Clearfil SE Bond (bond)	Identoflex composite polisher, polishing diamonds (40, 20 μm), coarse, medium, fine, superfine grits (Sof-Lex) Assessment: GC/MS, (UHP)LC-MS	Qualitative and quantitative (molecular weight, retention times, molecular and characteristic ions) analysis
Van Landuyt et al, 2012 In vitro Setting: composite sticks in silicon mold [limited clinical part]	Composite grinding (no water cooling); composite blocks in a silicon mold, plexiglass box 27 × 27 × 42 mm; air sampling 30 min 7 types of composites (n = 5/ per composite)	Filtek Supreme XTE nanocomposite Premise and Ceram.X and Tetric EvoCeram and Herculite nanohybrid	Grandia Direct Microhybrid Z-100 MP conventional hybrid	All samples ground with rough diamond bur [grain size: 100 μm] (micromotor) Assessment: TEM	Dust concentration Number and distribution of submicron particles
Van Landuyt et al, 2014 In vitro Setting: composite sticks in metal mold [limited clinical part]	Composite grinding (no water cooling); composite blocks in a metal mold; plexiglass box 270 × 270 × 420 mm; air sampling NR 5 types of composites (n = 5/per composite)	Filtek Supreme XTE nanocomposite GrandiO and Tetric EvoCeram nanohybrid	Grandia Direct Microhybrid Z-100 MP conventional hybrid	All samples ground with rough diamond bur [grain size: 100 μm] (micromotor) Assessment: mini DiSC; TEM; SMPS; ESP; EPR	Number and distribution of submicron particles Chemical identity of sampled particles Size distribution of composite dust OH-generation and non-specific surface activity index
Vig et al, 2019 RCT Setting: hospital orthodontic department	18 patients (6 per group); age NR; debonding procedures	Ceramic brackets with Transbond adhesive Ceramic adhesive pre-coated	Metal brackets with Transbond adhesive	All teeth ground with tungsten bur and slow-handpiece Assessment: (pDR)-1200 real-time monitor, Cascade impactor, SEM, EDX	Particulate concentration (respirable fraction)

BPA, bisphenol-A; DLS/ELS dynamic light scattering/electrophoretic light scattering; EDX, energy-dispersive x-ray spectroscopy; EPR, electron paramagnetic resonance spectroscopy; ESP, electrostatic precipitator; GC/MS, gas chromatography-mass spectrometry; HVE, high-volume evacuator; LC–MS/MS, liquid chromatography/mass spectroscopy; LDH, lactate dehydrogenase; Micro-ATR FTIR, micro-attenuated total reflectance Fourier transform infrared spectroscopy; NR, not reported; SEM, scanning electron microscopy; RCT, randomized controlled trial; SMPS, scanning mobility particle sizer; TEM, transmission electron microscopy; DiSi, diffusion size classifier.

**Table 2:** Risk of bias assessment of included studies (in vitro and in vivo) (*n* = 12).

Study	Baseline Similarity of Experimental Conditions (Selection Bias)	Blinding of Outcome Assessment (Detection Bias)	Incomplete Outcome Data (Attrition Bias)	Selective Reporting (Reporting Bias)	Other Bias
Bradna et al, 2017	low	unclear	low	unclear	low
Day et al, 2008	low	unclear	low	unclear	low
Cokic et al, 2016	low	unclear	low	unclear	low
Cokic et al, 2017	low	unclear	low	unclear	low
Cokic et al, 2019	low	unclear	low	unclear	low
Cokic et al, 2020	low	unclear	low	unclear	low
Gioka et al, 2009	low	unclear	low	unclear	low
Ireland et al, 2003	high	unclear	low	unclear	unclear
Johnston et al, 2009	low	unclear	low	unclear	low
Nilsen et al, 2019	low	unclear	low	unclear	low
Van Landuyt et al, 2012	low	unclear	low	unclear	low
Van Landuyt et al, 2014	low	unclear	low	unclear	low

**Table 3:** Risk of bias of included randomized clinical trials with the RoB 2.0 tool (*n* = 1).

Study	Randomization Process	Deviations from Intended Interventions	Missing Outcome Data	Measurement of the Outcome	Selection of the Reported Result	Overall
Vig et al, 2019	Low	Low	Low	Some concerns	Some concerns	Some concerns

**Table 4:** Quantitative data from meta-analyses and individual single studies for related comparisons and outcomes. The minus sign (−) shows larger effect for the second group under comparison. Bold indicate statistically significant comparisons.

#	Study ID	Comparison	Outcome	MD or SMD (95% CIs)	*p*-Value	Heterogeneity (I^2^%)
**1**	2 studies	Filtek Supreme XTE vs. Z100 MP	size% distribution of particles < 100 nm	SMD: 1.96 (0.85, 3.07)	**0.001**	0
		Filtek Supreme XTE vs. Tetric EvoCeram	size% distribution of particles < 100 nm	SMD: 1.62 (0.56, 2.68)	**0.003**	0
**2**	2 studies	Filtek Supreme XTE vs. GradiO	average particle number concentration (#/cm^3^ × 10^6^)	SMD: 0.24 (−0.75, 1.24)	0.63	0
**3**	2 studies	Filtek Supreme XTE vs. GradiO	% formazan reduction at 330–400 μg/mL (cell viability, WST-1 assay)	SMD: 0.19 (−0.17, 0.55)	0.30	69.0
**4**	Cokic 2019	Grinding of nanocomposite w/o water cooling vs. with water cooling (micromotor)	average particle number concentration (#/cm^3^ × 10^6^)	MD: 1.5 (1.2, 1.7)	**< 0.001**	−
**5**	Gioka 2009	CC vs. control	MCF-7 cell proliferation (% control)	MD: 60 (51.5, 68.5)	**< 0.001**	−
		LC vs. control	MCF-7 cell proliferation (% control)	MD: 28 (22.4, 33.6)	**< 0.001**	−
**6**	Cokic 2020	Transbond XT (orthodontic) vs. Supreme XTE	% formazan reduction at 400 μg/mL (cell viability, WST-1 assay) 24 h	MD: 6.8 (4.7, 8.9)	**< 0.001**	−
		Transbond XT (orthodontic) vs. GradiO	% formazan reduction at 400 μg/mL (cell viability, WST-1 assay) 24 h	MD: 10.6 (7.3, 13.9)	**< 0.001**	−
**7**	Cokic 2020	Transbond XT (orthodontic) vs. Filtek Supreme XTE	% cell membrane integrity reduction (LDH assay) 72 h	MD: 6.1 (2.5, 9.7)	**0.001**	−
		Transbond XT (orthodontic) vs. GradiO (nanohybrid)	% cell membrane integrity reduction (LDH assay) 72 h	MD: 10.9 (7.8, 14.0)	**< 0.001**	−

CC, chemically cured orthodontic adhesive; LC, light cured orthodontic adhesive; LDH, lactate dehydrogenase; MCF-7, human breast adenocarcinoma cell line; MD, mean difference; SMD, standardized mean difference; w/o, without; CIs, confidence intervals.

## Appendix A

**Table A1:** Detailed search strategy across databases.

No.	Electronic Database	Hits
1.	Medline via Pubmed	
	composite AND dust AND dent *	**51**
	((composite) OR (adhesive) OR (resin) OR (filler)) AND ((polishing) OR (grinding) OR (rotary instruments) OR (high speed handpiece) OR (slow speed handpiece) OR (high speed air turbine)) AND ((dental) OR (dentistry) OR (dent*)) AND ((aerosol) OR (suspended particle) OR (dust) OR (nano-dust) OR (nanodust) OR (airborne particle) OR (nanoparticle))	**141**
	(composite OR resin) AND (dust OR particl*) AND (orthodontic debond*)	**27**
2.	Scopus	
	composite AND dust AND dent *	**26**
	((composite) OR (adhesive) OR (resin) OR (filler)) AND ((polishing) OR (grinding) OR (rotary instruments) OR (high speed handpiece) OR (slow speed handpiece) OR (high speed air turbine)) AND ((dental) OR (dentistry) OR (dent*)) AND ((aerosol) OR (suspended particle) OR (dust) OR (nano-dust) OR (nanodust) OR (airborne particle) OR (nanoparticle))	**84**
	(composite OR resin) AND (dust OR particl*) AND (orthodontic debond*)	**23**
3.	Cochrane Central Register of Controlled Trials (CENTRAL)	
	composite AND dust AND dent*	**2**
	((composite) OR (adhesive) OR (resin) OR (filler)) AND ((polishing) OR (grinding) OR (rotary instruments) OR (high speed handpiece) OR (slow speed handpiece) OR (high speed air turbine)) AND ((dental) OR (dentistry) OR (dent*)) AND ((aerosol) OR (suspended particle) OR (dust) OR (nano-dust) OR (nanodust) OR (airborne particle) OR (nanoparticle))	**8**
	(composite OR resin) AND (dust OR particl*) AND (orthodontic debond*)	**6**
4.	Cochrane Database of Systematic Reviews (CDSR)	
	composite AND dust AND dent*	**0**
	((composite) OR (adhesive) OR (resin) OR (filler)) AND ((polishing) OR (grinding) OR (rotary instruments) OR (high speed handpiece) OR (slow speed handpiece) OR (high speed air turbine)) AND ((dental) OR (dentistry) OR (dent*)) AND ((aerosol) OR (suspended particle) OR (dust) OR (nano-dust) OR (nanodust) OR (airborne particle) OR (nanoparticle))	**0**
	(composite OR resin) AND (dust OR particl*) AND (orthodontic debond*)	**0**
5.	Open Grey	
	composite AND dust	**4**
6.	ClinicalTrials.gov (www.clinicaltrials.gov)	
	composite AND dust	**0**
7.	National Research Register (ISRCTN: www.controlled-trials.com)	
	composite AND dust	**1**
